# Supplementary material for: Accelerating Large-Scale-Structure data analyses by emulating Boltzmann solvers and Lagrangian Perturbation Theory
Source: Open Res Eur. 2022 Jun 15;1:152. Originally published 2021 Dec 16. [Version 2] doi: 10.12688/openreseurope.14310.2 (PMC10445901; doi:10.12688/openreseurope.14310.2)
Supplement: Peer review history

# Peer review: Reviewer response for version 2

**DOI:** 10.21956/openreseurope.16091.r29529

**Published:** 2022-07-20

**Authors:** Kazuya Koyama

**Affiliations:** 1 Institute of Cosmology and Gravitation, University of Portsmouth, Portsmouth, UK

The authors have made satisfactory revisions and I have no further comments.

Is the study design appropriate and does the work have academic merit?

Yes

Is the work clearly and accurately presented and does it cite the current literature?

Yes

If applicable, is the statistical analysis and its interpretation appropriate?

Yes

Are all the source data underlying the results available to ensure full reproducibility?

Yes

Are the conclusions drawn adequately supported by the results?

Yes

Are sufficient details of methods and analysis provided to allow replication by others?

Yes

Reviewer Expertise:

NA

I confirm that I have read this submission and believe that I have an appropriate level of expertise to confirm that it is of an acceptable scientific standard.

# Peer review: Reviewer response for version 1

**DOI:** 10.21956/openreseurope.15439.r29034

**Published:** 2022-04-29

**Authors:** Kazuya Koyama

**Affiliations:** 1 Institute of Cosmology and Gravitation, University of Portsmouth, Portsmouth, UK

This paper presents emulators for the linear power spectrum as well as components of Lagrangian Perturbation Theory (LPT). These linear emulators will play a crucial role as the major limitation of predicting non-linear power spectrum is indeed coming from the linear prediction from the Boltzmann code. The paper is well written and there is no major concern about the results. There are a few comments that may be useful.

 Although BAO peaks are well recovered by emulators, there are still residual errors for example in the suppression due to massive neutrinos. Can this be improved further by sampling k-bins more densely around BAO peaks?

 For the LPT emulator, it is good to see the accuracy of the final combined power spectrum with a set of reasonable bias parameters. Not all components will contribute equally, and it is good to know the accuracy of the final galaxy (and cross) power spectrum. This can be done by randomly sampling bias parameters with reasonable priors for example.

 For the mock cosmic shear analysis, it is useful to know a typical improvement on the MCMC run times using emulators as opposed to using CLASS. The difference between CLASS and CAMB for massive neutrinos is larger than emulation errors. It will be good to add a short discussion on why using CLASS is preferred to construct emulators.

Is the study design appropriate and does the work have academic merit?

Yes

Is the work clearly and accurately presented and does it cite the current literature?

Yes

If applicable, is the statistical analysis and its interpretation appropriate?

Yes

Are all the source data underlying the results available to ensure full reproducibility?

Yes

Are the conclusions drawn adequately supported by the results?

Yes

Are sufficient details of methods and analysis provided to allow replication by others?

Yes

Reviewer Expertise:

NA

I confirm that I have read this submission and believe that I have an appropriate level of expertise to confirm that it is of an acceptable scientific standard.

# Response

**Published:** 2022-06-09

**Authors:** Giovanni Arico'

**Affiliations:** Donostia International Physics Center, Spain

We thank the referee, Kazuya Koyama, for his careful reading of the paper and the comments raised. Following, we address his points:

1. As the referee noted, there are small residuals in the BAO peaks. We believe that these residuals are not due to poor sampling of the BAO scales (in the central panel of Fig. 3 we show indeed that the BAO scales are well sampled), but they are rather caused by the Principal Components Analysis (PCA) decomposition. In fact, while the first PCs describe the broad shape of the power spectrum, higher PCs are connected to the BAO oscillations. In this work, we have retained the first 64 PCs, which are sufficient to describe the broad shape of the power spectrum at 0.03%, but leave residuals at the BAO scales at 0.1% level. We have indeed chosen the number of PCs used in this work as a compromise between the accuracy of the decomposition and the complexity of the network emulation. Moreover, by adding more PCs we also include small-scale noise that the network will likely learn. In order to get accuracy below 0.1%, more PCs have to be included, at the price of a more complex training of the network (and noisier predictions). Nonetheless, if such precision will be required in the future, a better emulation can be achieved in this way. We have added this discussion to the paper.
2. We thank the referee for raising this point, it is indeed important to show a final accuracy for the combined cross spectra. We have added a Figure to our paper (Fig. 5) that shows the accuracy of the galaxy auto power spectrum and galaxy-matter cross power spectrum when drawing the bias parameters from realistic Lagrangian galaxy priors presented in Zennaro et al. 2021. We find a percent accuracy in both the power spectra at most of the scales considered.
3. In our case, when considering massless neutrinos (where the NN gain is effectively lower because Boltzmann solvers are faster), by using the NN emulator we reduced the analysis times by a factor between 10 and 100 with respect to CLASS. We stress that these factors are highly dependent on the setup (the algorithm used for sampling, machine employed, number of cores).
4. We showed that emulation techniques can be more accurate than the typical difference between CLASS and CAMB, arguably the two most used Boltzmann solvers. We highlight that even when dropping the fluid approximation of CLASS, we find a difference between the two codes of about 1% at the smallest scales considered (k = 50 h/Mpc ). Although such small scales are beyond the reach of the next generation LSS surveys, and therefore using either of the two Boltzmann solvers will likely produce the same outcome, we think that a more in-depth investigation of the differences between CLASS and CAMB within such a large cosmological space and wide range of scales is necessary. We leave such investigation for future works. We have added this discussion to the paper.

# Peer review: Reviewer response for version 1

**DOI:** 10.21956/openreseurope.15439.r28192

**Published:** 2022-01-04

**Authors:** Simeon Bird

**Affiliations:** 1 Department of Physics and Astronomy, Johns Hopkins University, Baltimore, MD, USA

The submission builds an emulator for the linear matter power spectrum. Although this can be computed analytically, so many computations are necessary that it may still be the limiting computational step in a likelihood evaluation and so this is a valuable thing to do. The authors show that their emulator, built from a neural network with ~200,000 training points, is very accurate (~0.1%). They also build an emulator for the LPT terms, which is slightly less accurate (0.5%) but still more so than the non-linear power spectra it should be compared to. They use a smaller training set for the LPT emulator, so achieving higher accuracy should not be difficult if desired. The authors make their code and dataset publicly available, the paper is well-written and I am happy to recommend that it pass peer review.

 I have only three minor comments on the text:

 Section 2.3:

 "Compared to Gaussian Processes, neural networks have the advantage of significantly lower computational and memory requirements, especially for large training sets."

 It is perhaps more accurate to say that neural networks have significantly better scaling with large training datasets than GPs. However, for small training sets GPs often train substantially faster than a neural network (ie, a GP built on this training set would be unlikely to take 24 hours to train).

 Section 3.2:

 " In each of these points, we compute the 15 Lagrangian cross power spectra as described above. As noted by Zennaro *et al*. (2021), some of these spectra can be negative. In these cases, we emulate the quantity:"

 Can the authors elaborate if they are emulating this quantity for all spectra or only for the negative spectra? If the latter (as I think from the text), could the authors explain how they avoid this confusing the emulator training? I suspect the answer is that the spectra are very rarely negative, and only very slightly so?

 Appendix A:

 The matter power spectrum with massive neutrinos differs a little more than the others between CLASS and CAMB. I have found a similar result and I think it is due to the fluid approximation CLASS uses for the neutrinos. I have been able to recover CAMB's results by increasing the CLASS accuracy parameters, at the cost of making the evaluation slower. I do not need the authors to do this, as I think their current setup is sufficiently accurate, I just note it for posterity.

Is the study design appropriate and does the work have academic merit?

Yes

Is the work clearly and accurately presented and does it cite the current literature?

Yes

If applicable, is the statistical analysis and its interpretation appropriate?

Yes

Are all the source data underlying the results available to ensure full reproducibility?

Yes

Are the conclusions drawn adequately supported by the results?

Yes

Are sufficient details of methods and analysis provided to allow replication by others?

Yes

Reviewer Expertise:

Cosmology, simulations, emulators.

I confirm that I have read this submission and believe that I have an appropriate level of expertise to confirm that it is of an acceptable scientific standard.

# Response

**Published:** 2022-04-20

**Authors:** Giovanni Arico'

**Affiliations:** Donostia International Physics Center, Spain

We thank the referee, Simeon Bird, for his careful reading of the manuscript and for his pointed comments, and apologise for the delay in the answer. We address his questions in what follows:

1. We agree with the referee that neural networks (NN) have better scaling than Gaussian Processes (GPs) in the training computational time. However, we note that in our experience, the memory requirements (for both model storage and training) of GPs are generally higher than neural networks, even with modest training sets. We will modify the text to specify this behaviour of NN and GPs.   
2. We thank the referee for pointing out this, probably the formulation "In this case" is a bit unfortunate, since it can lead to a misinterpretation of our procedure. We have a total of 15 cross-terms, some of which are always positive (specifically the terms 1-1, 1-delta, delta-delta, delta-delta^2, delta^2-delta^2, s^2-s^2, Laplacian delta-Laplacian-delta), whereas the others might be negative for some combination of the cosmological parameters. For each one of the latter terms, we always (i.e., for both positive and negative spectra) emulate the quantity P + |min(P)| +0.1, where |min(P)| is the absolute value of the minimum power spectrum measured for that term, considering all wave-numbers, redshifts and cosmological parameters in our training set. In this way, we avoid confusion in the emulation, since we always emulate the same quantity within a term. We will remove this ambiguity from the text.
3. We thank the referee for his specification, indeed we have obtained the results shown in the second and fourth panels of Figure 6 using the fluid approximation of CLASS. We have made some tests removing the fluid approximation, finding that it can only partially account for the difference between CLASS and CAMB. However, none of the cosmologies tested in the third panel of Figure 6 consider massive neutrinos, therefore all the differences between CAMB and CLASS in this case should come from their different dynamical dark energy implementations.

We plan to wait for the second referee report before uploading the new version of the text, to avoid confusion among different versions of the paper.
